# Supplementary material for: Multi‐dataset identification of innovative feature genes and molecular mechanisms in keratoconus
Source: J Cell Mol Med. 2024 Sep 19;28(18):e70079. doi: 10.1111/jcmm.70079 (PMC11412914; doi:10.1111/jcmm.70079)
Supplement: Supplementary file 2 — Material S2. [file JCMM-28-e70079-s002.zip › 7.GSEA analysis/1.ARL11/KEGG_UBIQUITIN_MEDIATED_PROTEOLYSIS.html]

Details for gene set KEGG\_UBIQUITIN\_MEDIATED\_PROTEOLYSIS[GSEA]

|  || Dataset | ARL11.group.cls#h\_versus\_l |
| Phenotype | group.cls#h\_versus\_l |
| Upregulated in class | h |
| GeneSet | KEGG\_UBIQUITIN\_MEDIATED\_PROTEOLYSIS |
| Enrichment Score (ES) | 0.14119451 |
| Normalized Enrichment Score (NES) | 0.8252901 |
| Nominal p-value | 0.67075664 |
| FDR q-value | 1.0 |
| FWER p-Value | 1.0 |
Table: GSEA Results Summary

  

Fig 1: Enrichment plot: KEGG\_UBIQUITIN\_MEDIATED\_PROTEOLYSIS      
 Profile of the Running ES Score & Positions of GeneSet Members on the Rank Ordered List

  

| SYMBOL | TITLE | RANK IN GENE LIST | RANK METRIC SCORE | RUNNING ES | CORE ENRICHMENT || 1 | MID1 | na | 640 | 0.447 | 0.0441 | Yes |
| 2 | KLHL13 | na | 1353 | 0.352 | 0.0729 | Yes |
| 3 | CBLB | na | 3964 | 0.202 | 0.0257 | Yes |
| 4 | SOCS3 | na | 4502 | 0.183 | 0.0358 | Yes |
| 5 | FANCL | na | 4860 | 0.170 | 0.0493 | Yes |
| 6 | UBE4B | na | 5723 | 0.144 | 0.0446 | Yes |
| 7 | HERC2 | na | 5781 | 0.143 | 0.0630 | Yes |
| 8 | UBA7 | na | 6191 | 0.132 | 0.0697 | Yes |
| 9 | ANAPC1 | na | 6781 | 0.117 | 0.0690 | Yes |
| 10 | CUL5 | na | 6817 | 0.116 | 0.0842 | Yes |
| 11 | UBE3C | na | 7484 | 0.102 | 0.0793 | Yes |
| 12 | SKP2 | na | 8159 | 0.089 | 0.0723 | Yes |
| 13 | DDB1 | na | 8232 | 0.088 | 0.0825 | Yes |
| 14 | RCHY1 | na | 8330 | 0.086 | 0.0917 | Yes |
| 15 | UBR5 | na | 8492 | 0.083 | 0.0987 | Yes |
| 16 | DDB2 | na | 8819 | 0.078 | 0.1001 | Yes |
| 17 | FZR1 | na | 9057 | 0.074 | 0.1036 | Yes |
| 18 | ERCC8 | na | 9202 | 0.072 | 0.1095 | Yes |
| 19 | CUL3 | na | 9531 | 0.067 | 0.1094 | Yes |
| 20 | PPIL2 | na | 9556 | 0.066 | 0.1180 | Yes |
| 21 | UBOX5 | na | 9952 | 0.061 | 0.1151 | Yes |
| 22 | TRIP12 | na | 9985 | 0.060 | 0.1226 | Yes |
| 23 | PRPF19 | na | 10399 | 0.055 | 0.1184 | Yes |
| 24 | MDM2 | na | 10671 | 0.052 | 0.1178 | Yes |
| 25 | UBA3 | na | 10905 | 0.049 | 0.1178 | Yes |
| 26 | NEDD4L | na | 11021 | 0.047 | 0.1211 | Yes |
| 27 | HUWE1 | na | 11125 | 0.046 | 0.1246 | Yes |
| 28 | VHL | na | 11342 | 0.043 | 0.1244 | Yes |
| 29 | MGRN1 | na | 11574 | 0.041 | 0.1235 | Yes |
| 30 | CDC23 | na | 11946 | 0.037 | 0.1179 | Yes |
| 31 | ANAPC4 | na | 12068 | 0.035 | 0.1193 | Yes |
| 32 | UBE2E1 | na | 12372 | 0.033 | 0.1151 | Yes |
| 33 | MAP3K1 | na | 12394 | 0.032 | 0.1191 | Yes |
| 34 | HERC4 | na | 12514 | 0.031 | 0.1200 | Yes |
| 35 | CBL | na | 12670 | 0.030 | 0.1197 | Yes |
| 36 | UBE2Q1 | na | 12841 | 0.028 | 0.1188 | Yes |
| 37 | UBE2O | na | 13522 | 0.023 | 0.1023 | Yes |
| 38 | UBE2L6 | na | 13560 | 0.022 | 0.1044 | Yes |
| 39 | ANAPC7 | na | 13750 | 0.021 | 0.1018 | Yes |
| 40 | UBE2QL1 | na | 13876 | 0.020 | 0.1010 | Yes |
| 41 | BIRC6 | na | 13888 | 0.020 | 0.1034 | Yes |
| 42 | XIAP | na | 14006 | 0.019 | 0.1027 | Yes |
| 43 | ANAPC5 | na | 14025 | 0.018 | 0.1047 | Yes |
| 44 | UBE2L3 | na | 14054 | 0.018 | 0.1065 | Yes |
| 45 | KEAP1 | na | 14056 | 0.018 | 0.1090 | Yes |
| 46 | RNF7 | na | 14240 | 0.017 | 0.1061 | Yes |
| 47 | RBX1 | na | 14278 | 0.017 | 0.1074 | Yes |
| 48 | UBE2Q2 | na | 14432 | 0.016 | 0.1051 | Yes |
| 49 | BIRC2 | na | 14603 | 0.014 | 0.1022 | Yes |
| 50 | UBE3B | na | 14916 | 0.012 | 0.0948 | Yes |
| 51 | NHLRC1 | na | 15190 | 0.010 | 0.0883 | Yes |
| 52 | UBE2NL | na | 15228 | 0.009 | 0.0886 | Yes |
| 53 | UBE2A | na | 15434 | 0.008 | 0.0838 | Yes |
| 54 | UBE2D2 | na | 15587 | 0.007 | 0.0803 | Yes |
| 55 | PIAS1 | na | 15768 | 0.005 | 0.0759 | Yes |
| 56 | CUL1 | na | 16128 | 0.003 | 0.0659 | Yes |
| 57 | FBXO2 | na | 16189 | 0.002 | 0.0645 | Yes |
| 58 | UBA1 | na | 16435 | 0.001 | 0.0575 | Yes |
| 59 | UBE3A | na | 16515 | 0.000 | 0.0552 | Yes |
| 60 | BTRC | na | 16988 | -0.003 | 0.0420 | Yes |
| 61 | FBXW8 | na | 17235 | -0.005 | 0.0356 | Yes |
| 62 | UBE2C | na | 17251 | -0.005 | 0.0359 | Yes |
| 63 | CDC16 | na | 17370 | -0.006 | 0.0333 | Yes |
| 64 | SKP1 | na | 17557 | -0.007 | 0.0289 | Yes |
| 65 | UBE2I | na | 17695 | -0.008 | 0.0261 | Yes |
| 66 | SAE1 | na | 17921 | -0.010 | 0.0210 | Yes |
| 67 | ANAPC11 | na | 17931 | -0.010 | 0.0221 | Yes |
| 68 | HERC1 | na | 17937 | -0.010 | 0.0233 | Yes |
| 69 | WWP1 | na | 17950 | -0.010 | 0.0243 | Yes |
| 70 | KLHL9 | na | 17971 | -0.010 | 0.0252 | Yes |
| 71 | RHOBTB2 | na | 18037 | -0.010 | 0.0248 | Yes |
| 72 | FBXO4 | na | 18039 | -0.010 | 0.0262 | Yes |
| 73 | PIAS3 | na | 18227 | -0.012 | 0.0224 | Yes |
| 74 | UBE2G2 | na | 18272 | -0.012 | 0.0229 | Yes |
| 75 | NEDD4 | na | 18307 | -0.012 | 0.0236 | Yes |
| 76 | FBXW11 | na | 18745 | -0.015 | 0.0131 | Yes |
| 77 | BRCA1 | na | 18769 | -0.015 | 0.0146 | Yes |
| 78 | UBE2J1 | na | 18872 | -0.016 | 0.0139 | Yes |
| 79 | CUL4A | na | 18922 | -0.016 | 0.0148 | Yes |
| 80 | SYVN1 | na | 19170 | -0.018 | 0.0102 | Yes |
| 81 | ANAPC2 | na | 19314 | -0.019 | 0.0088 | Yes |
| 82 | WWP2 | na | 19692 | -0.022 | 0.0009 | Yes |
| 83 | STUB1 | na | 19964 | -0.023 | -0.0036 | Yes |
| 84 | UBE2J2 | na | 20105 | -0.024 | -0.0043 | Yes |
| 85 | CBLC | na | 20116 | -0.024 | -0.0011 | Yes |
| 86 | UBE2U | na | 20197 | -0.025 | 0.0001 | Yes |
| 87 | FBXW7 | na | 20279 | -0.026 | 0.0013 | Yes |
| 88 | UBE4A | na | 20313 | -0.026 | 0.0040 | Yes |
| 89 | UBE2K | na | 20365 | -0.026 | 0.0063 | Yes |
| 90 | ANAPC13 | na | 20443 | -0.027 | 0.0078 | Yes |
| 91 | UBE2W | na | 20475 | -0.027 | 0.0107 | Yes |
| 92 | TRIM37 | na | 20505 | -0.028 | 0.0138 | Yes |
| 93 | TRAF6 | na | 21028 | -0.032 | 0.0031 | Yes |
| 94 | CUL4B | na | 21172 | -0.033 | 0.0035 | Yes |
| 95 | UBE2F | na | 21574 | -0.036 | -0.0030 | Yes |
| 96 | PML | na | 21643 | -0.036 | 0.0001 | Yes |
| 97 | UBE2Z | na | 21657 | -0.037 | 0.0048 | Yes |
| 98 | CDC27 | na | 21812 | -0.038 | 0.0056 | Yes |
| 99 | BIRC3 | na | 21919 | -0.039 | 0.0080 | Yes |
| 100 | PIAS2 | na | 22018 | -0.040 | 0.0108 | Yes |
| 101 | DET1 | na | 22027 | -0.040 | 0.0162 | Yes |
| 102 | CUL7 | na | 22033 | -0.040 | 0.0216 | Yes |
| 103 | AIRE | na | 22124 | -0.041 | 0.0248 | Yes |
| 104 | CUL2 | na | 22180 | -0.042 | 0.0290 | Yes |
| 105 | UBE2D4 | na | 23081 | -0.051 | 0.0102 | Yes |
| 106 | UBE2E3 | na | 23319 | -0.054 | 0.0108 | Yes |
| 107 | TRIM32 | na | 23591 | -0.057 | 0.0110 | Yes |
| 108 | PIAS4 | na | 23627 | -0.058 | 0.0180 | Yes |
| 109 | SMURF1 | na | 23663 | -0.058 | 0.0251 | Yes |
| 110 | CDC20 | na | 23671 | -0.058 | 0.0331 | Yes |
| 111 | ANAPC10 | na | 23673 | -0.058 | 0.0412 | Yes |
| 112 | CDC26 | na | 23700 | -0.058 | 0.0486 | Yes |
| 113 | UBE2S | na | 23841 | -0.060 | 0.0530 | Yes |
| 114 | UBE2G1 | na | 24005 | -0.062 | 0.0570 | Yes |
| 115 | ITCH | na | 24078 | -0.063 | 0.0638 | Yes |
| 116 | UBE2R2 | na | 24356 | -0.067 | 0.0652 | Yes |
| 117 | CDC34 | na | 24505 | -0.069 | 0.0706 | Yes |
| 118 | SMURF2 | na | 24679 | -0.072 | 0.0757 | Yes |
| 119 | UBA2 | na | 24881 | -0.074 | 0.0803 | Yes |
| 120 | UBE2D1 | na | 24889 | -0.075 | 0.0905 | Yes |
| 121 | SIAH1 | na | 25145 | -0.078 | 0.0941 | Yes |
| 122 | UBE2D3 | na | 25337 | -0.081 | 0.0999 | Yes |
| 123 | UBE2M | na | 25668 | -0.085 | 0.1022 | Yes |
| 124 | UBE2B | na | 25803 | -0.087 | 0.1106 | Yes |
| 125 | UBE2N | na | 25867 | -0.088 | 0.1211 | Yes |
| 126 | UBE2E2 | na | 26618 | -0.101 | 0.1135 | Yes |
| 127 | SOCS1 | na | 26619 | -0.101 | 0.1277 | Yes |
| 128 | UBE2H | na | 26643 | -0.101 | 0.1412 | Yes |
| 129 | UBA6 | na | 27912 | -0.129 | 0.1226 | No |
| 130 | HERC3 | na | 34495 | -0.531 | 0.0067 | No |
Table: GSEA details [plain text format]

  

Fig 2: KEGG\_UBIQUITIN\_MEDIATED\_PROTEOLYSIS      
 Blue-Pink O' Gram in the Space of the Analyzed GeneSet

  

Fig 3: KEGG\_UBIQUITIN\_MEDIATED\_PROTEOLYSIS: Random ES distribution      
 Gene set null distribution of ES for **KEGG\_UBIQUITIN\_MEDIATED\_PROTEOLYSIS**
